# Supplementary material for: High circulating elafin levels are associated with Crohn’s disease-associated intestinal strictures
Source: PLoS One. 2020 Apr 14;15(4):e0231796. doi: 10.1371/journal.pone.0231796 (PMC7156098; doi:10.1371/journal.pone.0231796)
Supplement: S1 Table — (PDF) [file pone.0231796.s001.pdf]

S1 Table

Baseline Characteristics

Table 1	cohort 1			cohort 2		
Baseline Characteristics	healthy	UC	CD	healthy	UC	CD
Elafin Range (ng/mL)	2.9-38	5.2-55	3.3-39	4.2-20	4.1-56	3.1-32
Age at Collection (mean ± SEM)	46±2	40±2	35±2	43±3	37±1	38±2
Age at diagnosis (mean ± SEM)		35±2	30±2		28±1	25±1
Duration of Disease in Years (mean ± SEM)		6±1	6±1		8±1	13±2
Gender (% Male)	42	35	25	40	56	52
Percentage Who Used Biologics		13	38		16	45
Percentage Who Used Steroids		17	52		32	30
Percentage Who Used Immunomodulators		9	14		21	38
Percentage Who Used 5-aminosalicyclic acid (5-ASA)		74	24		58	24
Percentage of Current Smoker (%)	20	13	21	20	7	12
C-reactive protein (CRP) Levels (mg/L) (mean ± SEM)		1.18 ±0.42	4.77 ±1.43		0.75±0.16	1.59±0.39
UC Partial Mayo Score (mean ± SEM)		2.09 ±0.47			3.04±0.29	
UC Mayo Endoscopic Score (mean ± SEM)		1.64 ±0.19			1.13±0.14	
UC Partial Mayo Score 6-18 months later (mean ± SEM)		2.40 ±0.52			1.59±0.28	
UC Percentage of ulcerative proctitis (%)		38			16	
UC Percentage of proctosigmoiditis (%)		10			0	
UC Percentage of Left sided colitis (%)		24			59	
UC Percentage of pancolitis (%)		29			22	
CD Harvey Bradshaw Index (mean ± SEM)			3.67 ±0.77			4.22±0.44
CD Harvey Bradshaw Index 6-18 months later (mean ± SEM)			2.54 ±0.65			1.38±0.26
**Montreal Classification of CD**						
CD Age at diagnosis less than 16 years A1 %			12			27
CD Age at diagnosis 17-40 years A2 %			59			60
CD Age at diagnosis over 40 years A3 %			29			12
CD ileal L1 %			56			23
CD colonic L2 %			6			15
CD ilealcolonic L3 %			38			63
CD isolated upper digestive tract L4 %			13			7
CD non-stricturing, non-penetrating B1 %			43			57
CD stricturing B2 %			50			24
CD penetrating B3 %			11			27
CD perianal disease P %			11			0
n	50	23	28	20	57	67
