# Supplementary material for: High circulating elafin levels are associated with Crohn’s disease-associated intestinal strictures
Source: PLoS One. 2020 Apr 14;15(4):e0231796. doi: 10.1371/journal.pone.0231796 (PMC7156098; doi:10.1371/journal.pone.0231796)
Supplement: S2 Table — (PDF) [file pone.0231796.s002.pdf]

S2 Table

A Disease locations and medication uses of UC patients

Percentage of medication uses(%)		E1	E2	E3	Biologics	steroid	immunomodulators	5-ASA
**Elafin<18000pg/ml**	**count**	17	24	15	12	12	12	42
	**Percentage(%)**	27.87	39.34	24.59	19.67	19.67	19.67	62.69
**Elafin>=18000pg/ml**	**count**	3	11	5	2	10	3	11
	**Percentage(%)**	13.04	48.00	21.74	8.00	40.00	12.00	44.00

B Disease locations of CD patients

Percentage of medication uses(%)		A1	A2	A3	L1	L2	L3	L4	B1	B2	B3	P
**Elafin<8000pg/ml**	**count**	2	34	5	15	5	20	2	31	10	2	5
	**Percentage(%)**	4.88	82.93	11.63	34.88	11.63	46.51	4.88	75.61	24.39	4.88	11.63
**Elafin>=8000pg/ml**	**count**	11	27	8	12	7	26	3	23	21	5	10
	**Percentage(%)**	23.91	62.79	17.39	26.09	15.22	56.52	6.52	50	45.65	10.87	21.74

C Medication uses of CD patients

Percentage of medication uses(%)		Biologics	steroid	immunomodulators	5-ASA
**Elafin<8000pg/ml**	**count**	16	19	13	10
	**Percentage(%)**	39.02	46.34	31.71	24.39
**Elafin>=8000pg/ml**	**count**	23	18	17	12
	**Percentage(%)**	50.00	39.13	36.96	26.09

D Age in IBD patients

Age in CD patients

	mean	sem	n
non-stricturing CD	35	1.63	45
stricturing	37	2.41	20
	n.s. CDNS vs CDS		
Elafin >8000pg/ml, non-stricturing CD	35	2.66	15
Elafin <8000pg/ml, non-stricturing CD	36	2.07	30
	n.s. high vs low elafin CDNS		
Elafin >8000pg/ml, stricturing CD	37	3.10	13
Elafin <8000pg/ml, stricturing CD	35	4.04	7
	n.s. high vs low elafin CDS		

Age in UC patients

	mean	sem	n
Elafin <18000pg/ml	39.4	1.5	60
Elafin >18000pg/ml	40.4	3.2	24
	n.s.		

E BMI in IBD patients

BMI in CD patients

	mean	sem	n
non-stricturing CD	27	0.56	45
stricturing CD	26	2.06	20
	n.s.		
Elafin >8000pg/ml, non-stricturing CD	26	2.08	15
Elafin <8000pg/ml, non-stricturing CD	27	0.90	30
	n.s.		
Elafin >8000pg/ml, stricturing CD	28	2.61	13
Elafin <8000pg/ml, stricturing CD	21	2.00	7
	n.s.		

BMI in UC patients

	mean	sem	n
>18000pg/ml	26.6	2.94	60
<18000pg/ml	25.7	1.20	24
	n.s.		

F Duration of disease in IBD patients

Duration of diseases (Years) in CD patients

	mean	sem	n
non-stricturing CD	3.4	0.58	45
stricturing	5.5	0.78	20
	p=0.0425 CDNS vs CDS		
Elafin >8000pg/ml, non-stricturing CD	4.5	1.1	15
Elafin <8000pg/ml, non-stricturing CD	2.7	0.7	30
	n.s. high vs low elafin CDNS		
Elafin >8000pg/ml, stricturing CD	8.5	0.6	13
Elafin <8000pg/ml, stricturing CD	3.0	1.1	7
	p=0.0001 high vs low elafin CDS		

Duration of Disease (Years) in UC patients

	mean	sem	n
Elafin <18000pg/ml	7.6	0.9	60
Elafin >18000pg/ml	8.8	1.6	24
	n.s.		
