# Supplementary material for: High circulating elafin levels are associated with Crohn’s disease-associated intestinal strictures
Source: PLoS One. 2020 Apr 14;15(4):e0231796. doi: 10.1371/journal.pone.0231796 (PMC7156098; doi:10.1371/journal.pone.0231796)
Supplement: S3 Table — (PDF) [file pone.0231796.s003.pdf]

S3 Table

A Baseline characteristics

Colonic tissues	non IBD	UC	CD without stricture	CD with stricture
Elafin mRNA Expression (fold)	5.7±1.89	11.8±2.67	5.4±1.0	2.9±2.3
Age at Collection (Mean±SEM)	62±2.2	43±2.1	45±3.6	36±6.1
Gender (% Male)	73	55	62	50
Histology Score (Mean±SEM)	2.6±0.3	7.5±0.4	8.8±0.7	8.6±1.2
Simple colitis activity score	N/A	6.8±0.6	N/A	N/A
HBI	N/A	N/A	7.4±1.2	5.0±0.5
% of biologics	0	24	50	40
% of 6MP or steroid	0	51	66	33
Duration of disease (years)	26±3	12±2	8±3	18±3
n	40	52	28	15

B

UC patients

Colonic elafin mRNA expresson	% Current anti-TNF treatment	% Current 6-MP or steroid	% Male sex	BMI	Age	Duration of disease (Years)	n
mean							
low tertile (0-1.05 fold)	28.57	57.14	52.94	22.5	38.1	8.3	18
middle terile (1.11-4.94 fold)	25.00	56.25	66.67	23.1	45.5	18.5	16
high (5.80-75 fold)	18.75	37.50	37.50	22.1	45.9	13.1	18
sem							
low tertile (0-1.05 fold)				0.88	3.70	1.0	
middle terile (1.11-4.94 fold)				1.04	3.49	4.3	
high (5.80-75 fold)				0.85	3.08	2.8	
				n.s.	n.s.	n.s.	

C

CD patients

Colonic Elafin mRNA expression	% Current anti-TNF treatment	% Current 6-MP or steroid	% Male Sex	BMI	age	Duration of disease (Years)	n
mean							
low terile (0-0.6 fold)	50.00	70.00	63.3	25.03	45.42	13	15
middle tertile (0.8-4.1)	14.29	35.71	61.5	24.57	42.69	20	14
high terile (4.2-14.6 fold)	38.46	61.54	66.7	23.48	43.42	17	14
sem							
low terile (0-0.6 fold)				1.04	4.08	3	
middle tertile (0.8-4.1)				1.61	3.71	4	
high terile (4.2-14.6 fold)				1.17	3.27	3	
				n.s.	n.s.	n.s.	
mean							
CD with stricture	30.0	70	83.3	25.03	45.42	13	15
CD without stricture	30.8	50	64.0	24.07	43.04	18	28
sem							
CD with stricture				1.04	4.08	3	
CD without stricture				0.97	2.38	2	
				n.s.	n.s.	n.s.	
