# Supplementary figures and images for: High circulating elafin levels are associated with Crohn’s disease-associated intestinal strictures
Source: PLoS One. 2020 Apr 14;15(4):e0231796. doi: 10.1371/journal.pone.0231796 (PMC7156098; doi:10.1371/journal.pone.0231796)

S1 Figure

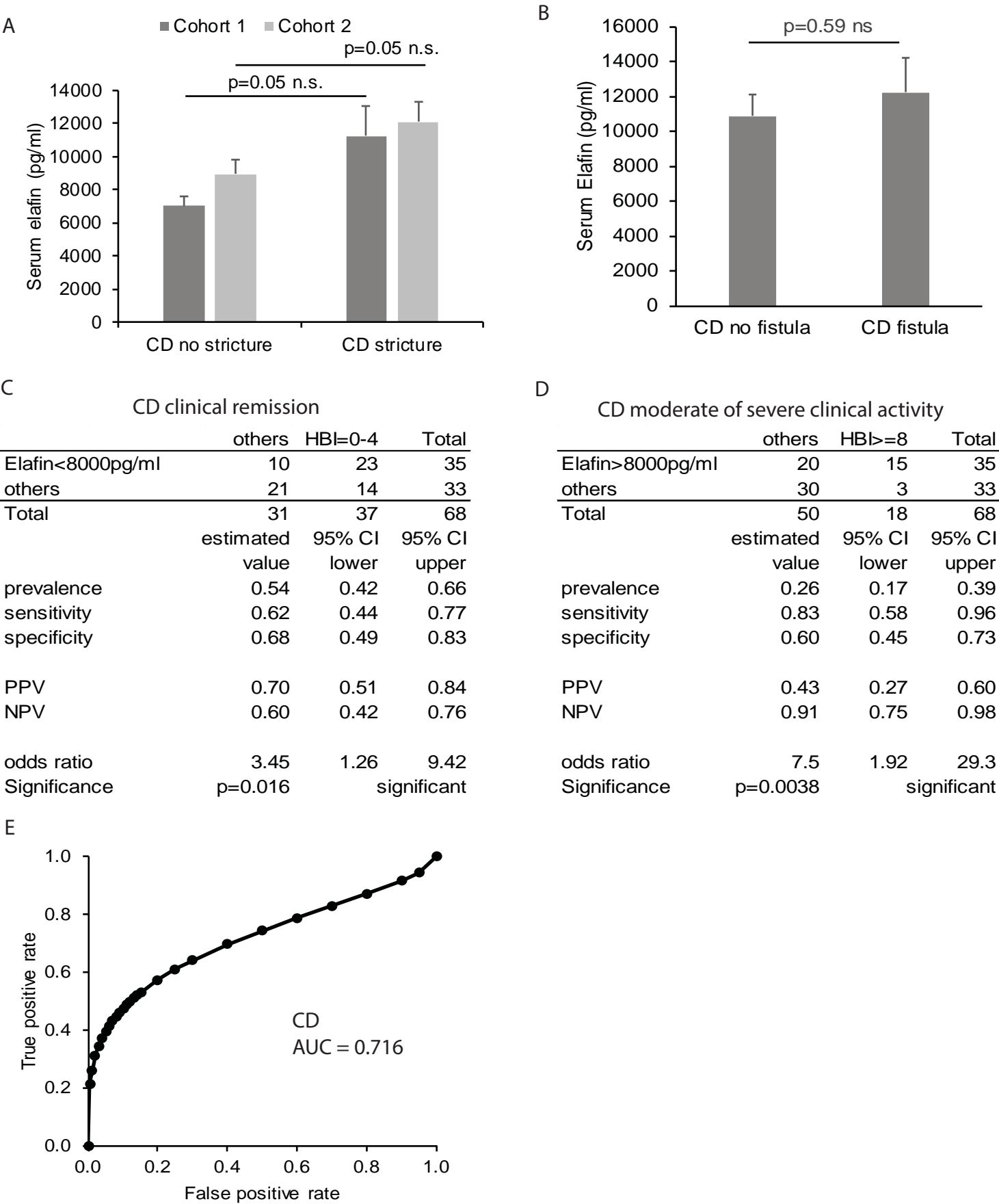

Supplement: S1 Fig — (A) The stricturing CD patients had higher serum elafin levels than non-stricturing CD patients in two separate datasets from cohorts 1 and 2. The differences were statistically insignificant. Two-group comparison was done by Student’s t-test. (B) Serum elafin levels in 18 CD patients with intestinal fistulas versus 67 CD patients without intestinal fistulas in a combined dataset. The difference was statistically insignificant. (C-D) Prevalence, sensitivity, specificity, positive predictive value, negative predictive value, and odds ratio values of elafin test in indicating (C) CD clinical remission and (D) moderate or severe CD clinical activity. (E) ROC curve with AUC value demonstrates the moderate accuracy of using the elafin test for indicating CD clinical disease activity. Optimal cutoff point is 8000pg/ml. (PDF) [file pone.0231796.s004.pdf]

S2 Figure

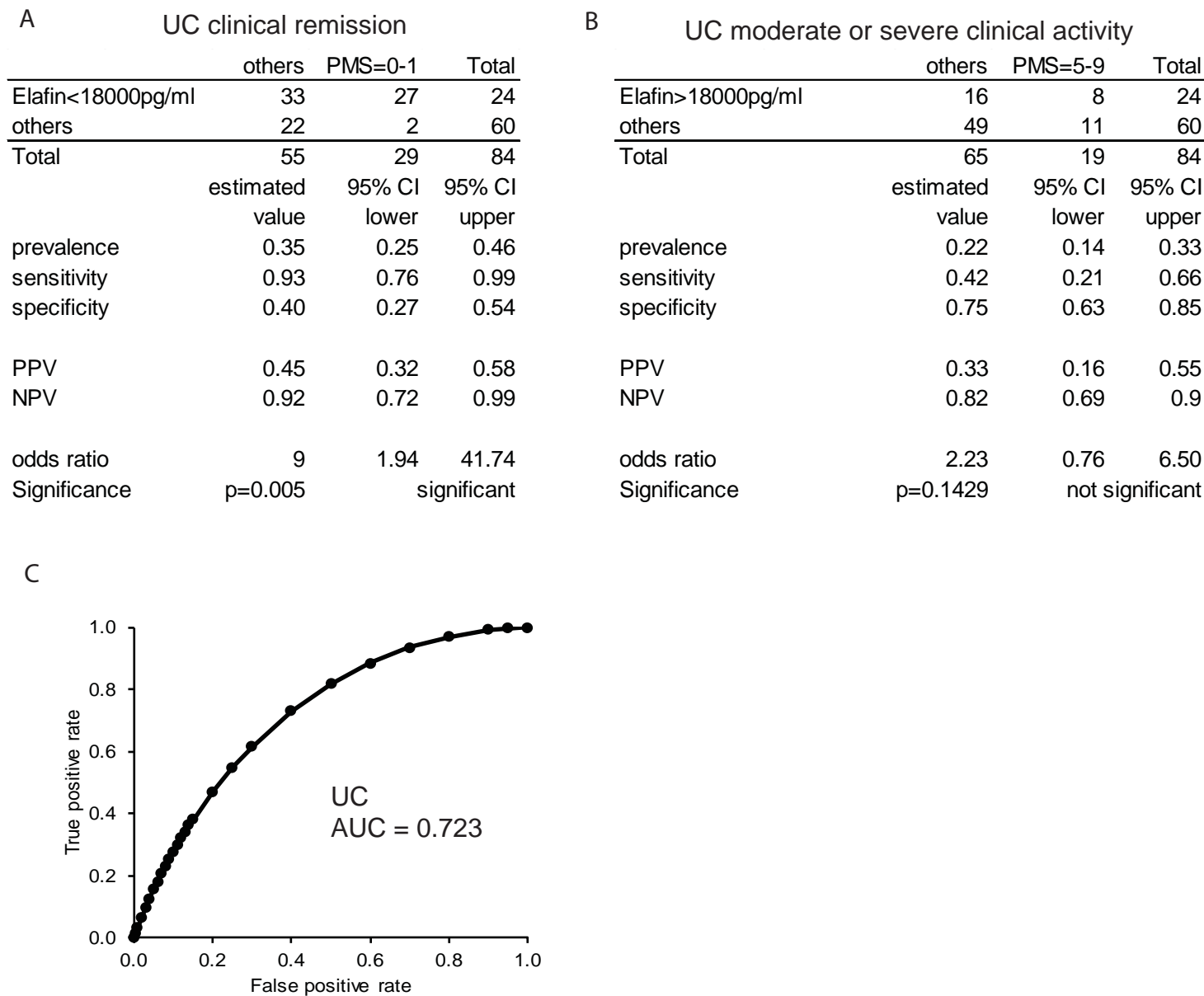

Supplement: S2 Fig — (A-B) Prevalence, sensitivity, specificity, positive predictive value, negative predictive value, and odds ratio values of elafin test in indicating (A) UC clinical remission and (B) moderate or severe UC clinical activity. (C) ROC curve with AUC value demonstrates the moderate accuracy of using the elafin test for indicating UC clinical disease activity. Optimal cutoff point is 18000pg/ml. (PDF) [file pone.0231796.s005.pdf]

S3 Figure

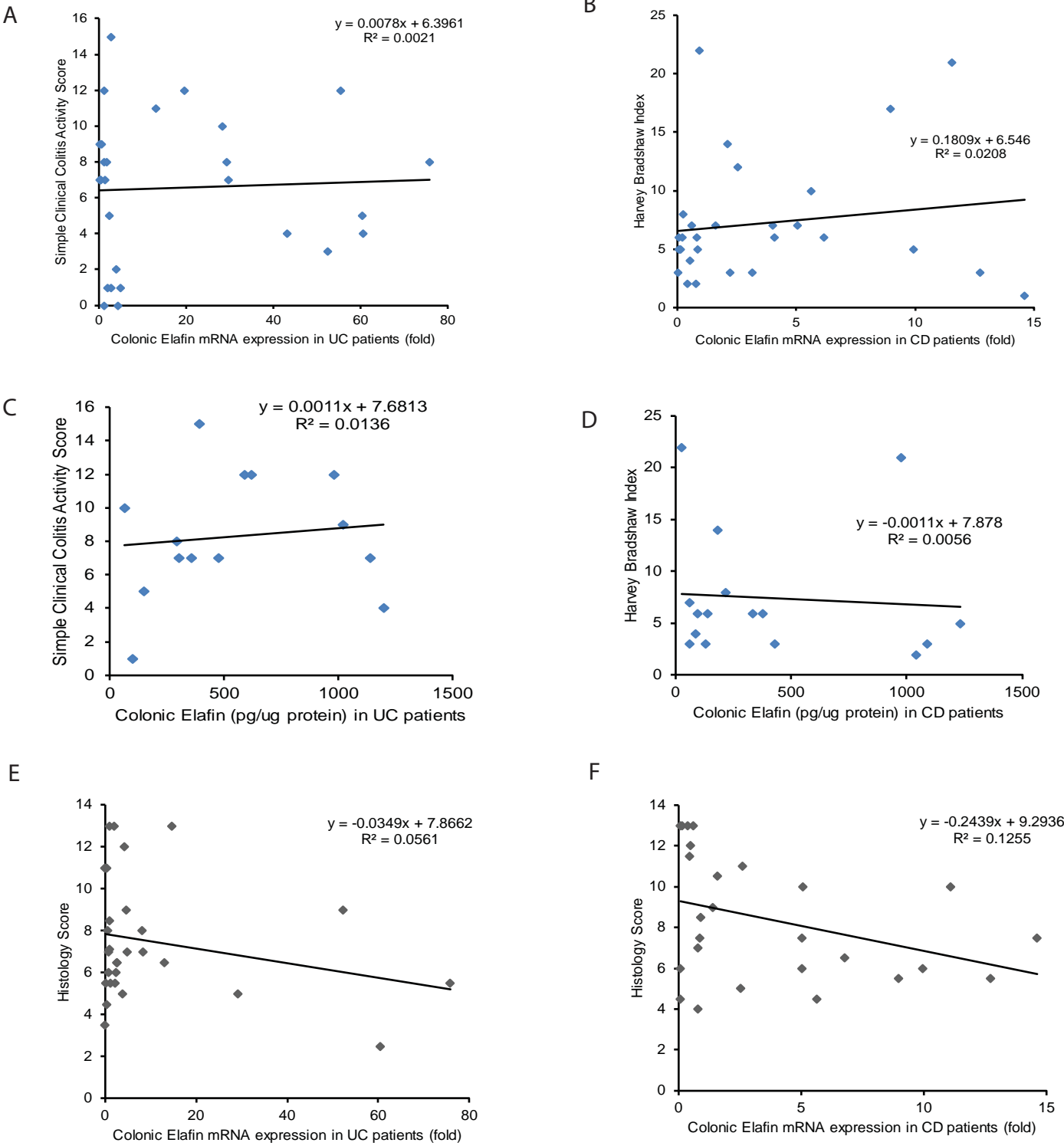

Supplement: S3 Fig — (A-B) Scatter plots show no significant correlation between clinical disease activity and colonic elafin mRNA expression in UC and CD patients. (C-D) Scatter plots show no significant correlation between clinical disease activity and colonic elafin protein expression in 30 UC and 27 CD patients. Simple Clinical Colitis Activity Score for UC patients. Harvey Bradshaw Index for CD patients. (E-F) Scatter plots show the weak negative correlation between colonic histology score and colonic elafin mRNA expression in 30 UC and 27 CD patients. The analysis included 26 UC patients and 29 CD patients. (PDF) [file pone.0231796.s006.pdf]

S5 Figure

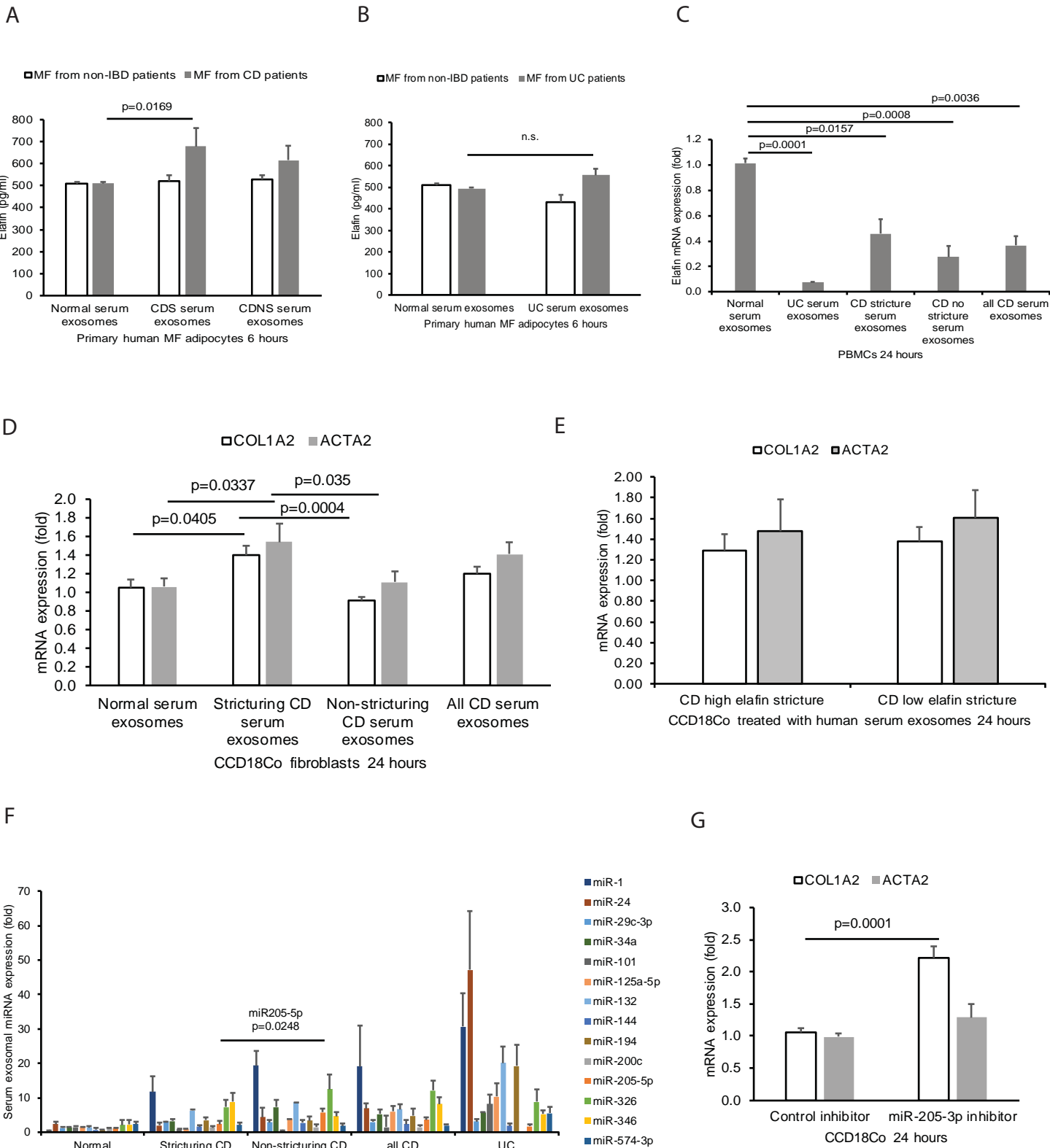

Supplement: S5 Fig — (A) Serum-starved primary human mesenteric fat adipocytes were exposed to 100μg/ml serum exosomes from normal, stricturing CD (CDS), or non-stricturing CD (CDNS) patients for 16 hours, followed by incubation with serum-free DMEM media for 6 hours. (B) Serum-starved primary human mesenteric fat adipocytes were exposed to 100μg/ml serum exosomes from normal or UC patients for 16 hours, followed by incubation with serum-free DMEM media for 6 hours. Conditioned media were collected from elafin ELISA. Each adipocyte group consisted of 5 patients. (C) PBMCs were exposed to 100μg/ml serum exosomes normal, stricturing CD, non-stricturing CD, and UC patients for 24 hours. (D-E) The human intestinal fibroblasts were incubated with 100μg/ml of human serum exosomes in serum-free DMEM for 24 hours. The collagen (COL1A2) mRNA expression was determined by real-time RT-PCR. Each serum exosome treatment group consisted of 6 patients per group. Multiple group comparison was done by one-way ANOVA. (E) The human colonic CCD-18Co fibroblasts were incubated with 100μg/ml of human serum exosomes from high elafin CD group (>8000pg/ml) and low elafin CD group (<8000pg/ml) in serum-free DMEM for 24 hours. Each serum exosome treatment group consisted of 6 patients per group. (F) Serum exosomal miRNA expression was determined by real-time RT-PCR. (G) Serum-starved CCD-18Co fibroblasts were treated with miR205-5p power inhibitor for 24 hours. Collagen (COL1A2) and ACTA2 mRNA expression were determined by real-time RT-PCR. (PDF) [file pone.0231796.s008.pdf]
