# Supplementary material for: High circulating elafin levels are associated with Crohn’s disease-associated intestinal strictures
Source: PLoS One. 2020 Apr 14;15(4):e0231796. doi: 10.1371/journal.pone.0231796 (PMC7156098; doi:10.1371/journal.pone.0231796)
Supplement: S4 Fig — (A) Colonic COL1A2 and elafin mRNA expression were determined by real-time RT-PCR and four samples were selected for RNA sequencing. The colonic tissues from stricturing CD patients had high collagen and low elafin mRNA expression. (B) Heat-map of increased (green) and decreased (red) gene expression in the colonic tissues of 2 stricturing CD patients versus 2 non-stricturing CD patients. The RNA-Seq was performed by Omega Biosciences. (C) A list of overexpressed and underexpressed genes in the colonic tissues of CDS patients, compared to CDNS patients. 2 CD patients (HBI = 2) per group. >20-fold increased and >9-fold decreased genes in log2(fold change) were shown. (PDF) [file pone.0231796.s007.pdf]

S4 Figure

A

Colonic tissue sample ID	COL1A2 mRNA expression (fold)	Elafin mRNA expression (fold)	
11-106	2.07	0.00	CDS in RNA-seq
10-34	5.94	0.10	CDS in RNA-seq
M9 C12-4593	0.21	4.37	CDNS in RNA-seq
C12-4593	0.28	3.80	CDNS in RNA-seq

B

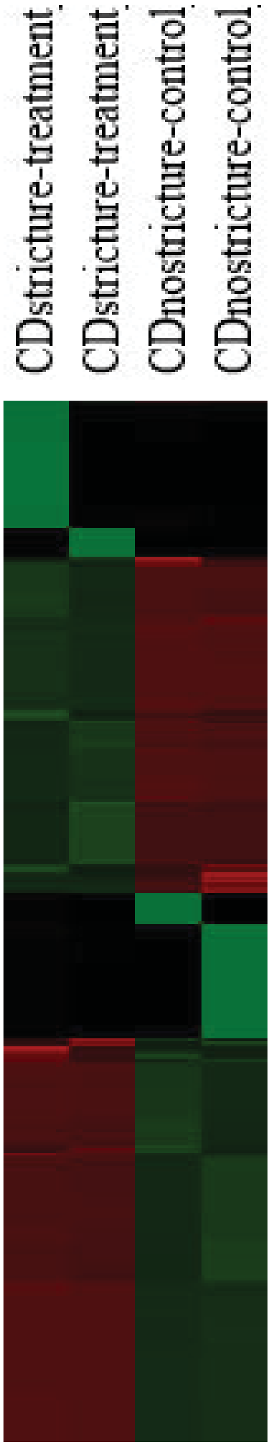

C

Consistenly increased mRNAs in stricturing CD				
ACTA2-AS1	ENG	LOC100652999	PSPC1	TUBGCP4
ADCY9	EPHA5-AS1	LOC101927751	PTPN14	VAT1
ADNP	ERCC6L	LOC101927827	RAB3GAP2	VIM-AS1
ADPRM	ERICH1	LOC105369332	RARB	ZBTB12
AFG3L2	EVA1B	LOC389199	RBBP4	ZC2HC1C
AGO2	FAM198B-AS1	LOC440570	RCC1L	ZC3H14
AHSA2P	FAM45BP	LOC440934	REV3L	ZEB1
ANKRD34A	FHOD3	LPIN3	RIC1	ZNF106
APMAP	FLG	LRRC37A4P	RIOX2	ZNF142
APOL1	FLNA	LTBP2	RNF217	ZNF154
ARSG	FN1	M6PR	RNF219-AS1	ZNF227
ASAP2	GDF7	MAP7D1	RUVBL1	ZNF32-AS1
ASB8	GEMIN7	MED12L	SARM1	ZNF483
ATG9B	GIGYF1	MED19	SCN8A	ZNF542P
ATP6V1FNB	GNPDA2	MEF2D	SHPRH	ZNF550
ATXN1	GNRHR2	MIA3	SLC16A6	ZNF551
AUNIP	GPR17	MIR1-1HG	SLC19A1	ZNF814
B4GALT2	GPR39	MMRN2	SLC24A5	
BMP2K	HAGLR	MPPE1	SLC2A4RG	
C12orf65	HARS	MRPL19	SLF2	
C1RL	HLA-DQB1-AS1	MYO5B	SMG6	
C22orf23	HLA-L	NAV1	SMIM15	
C8orf88	HOTAIRM1	NAV2	SNRNP35	
C9orf131	HOTS	NCBP1	SPEG	
CADM3-AS1	HOXD8	NCBP2	SPIDR	
CALD1	HPS3	NECAP2	SPIRE1	
CCDC25	HRNR	NEFM	STBD1	
CCDC66	HTRA2	NEXN-AS1	STK24	
CDAN1	HYAL1	NODAL	SUMF2	
CDC40	IGF2	NOP9	SUPT20HL2	
CDR1	IGHMBP2	NR4A1	SURF4	
CELSR2	IL17D	NUDT6	SUSD5	
CFAP44	IL21R	OGFR	SVIL-AS1	
CHORDC1	ILK	PACRGL	SYNM	
CIPC	IRF2BP2	PAIP2	TBCD	
CLBA1	JADE1	PAPLN	TCEAL3	
CLDN22	KIAA1614-AS1	PCBP2	TCF7L2	
CNN1	KRAS	PCSK7	TCOF1	
CNNM3	LDB3	PCYT2	TEX10	
COL1A2	LDLRAD2	PHLDA1	TFDP2	
COPG2	LETM2	PHYHIPL	TMED7	
CPT2	LINC00319	PIGK	TMEM181	
CREB3	LINC00602	PLEKHM2	TMEM252	
CREM	LINC01279	POLR2B	TNFRSF6B	
CRMP1	LOC100101148	PPM1A	TOMM34	
DNAJC9-AS1	LOC100128006	PPM1L	TRNP1	
DSE	LOC100128494	PRICKLE2-AS1	TSIX	
EFCAB13	LOC100506071	PRPF31	TSR2	
EIF4H	LOC100506282	PRR29	TSTD2	
ENAH	LOC100507507	PRRT3	TTC23	

Consistently decreased mRNAs in stricturing CD							
ABHD11	CAPN1	DNM2	HIPK2	LOC102724814	PAGR1	SH3BGRL2	UBE2J1
ABHD2	CAPN12	DNMT1	HK2	LOC103171574	PARM1	SHROOM3	UBE2K
ABO	CASP1	DSC2	HNF1A	LOC105373609	PBLD	SLC1A4	UBE2R2
ADAM22	CCDC12	DSP	HNF4A	LOC105374454	PCGF2	SLC26A3	UQCC3
ADCY3	CD177	DUOXA1	HOXA10	LOC145694	PCNT	SLC27A1	USP30
AGAP2	CEACAM1	EEF2KMT	HOXA11	LOC646626	PDE1C	SLC30A1	USP32
AK3	CEACAM5	EFNB1	HOXA11-AS	LOC652276	PHGR1	SLC35F5	VASN
AKNA	CEACAM6	EGLN1	HPCAL1	LRIT2	PI3	SLC37A1	VAV1
AKR1C3	CEACAM7	EHBP1L1	HPS4	LRRC37A	PIAS1	SLC39A4	VDR
ALDH1A3	CES2	EHMT2	ILF2	LSP1	PIGR	SLC39A7	VIL1
ANPEP	CHAF1A	EI24	INF2	LSR	PIK3R3	SLC9A3-AS1	WTAP
ANXA11	CHCHD10	EIF2S3B	INKA2	MAGEA5	PLA2G2A	SMAD1-AS1	YIF1B
ANXA2P1	CHGA	ELP5	IRF8	MAPK3	PLK1	SMARCA4	YWHAE
AOC1	CHGB	EML4	IRGQ	MARVELD3	PODNL1	SNTB1	ZBTB7B
AP1B1	CIZ1	EPB41L3	ITCH	MAST4	POLR2A	SNX22	ZC3H12B
AP1G2	CKMT1A	EPCAM	ITM2C	MBD2	POLR2H	SOS1	ZNF148
AP3S1	CLCA1	EPS15	KDM3B	MED14OS	POLR2J	SPACA4	ZNRF1
APOL6	CLCA4	FABP1	KHDC1	METRN	POLR3H	SPAG5-AS1	
ARFGAP2	COPZ1	FAM120B	KLHDC2	MIDN	POR	SPATS2	
ARHGDIA	CORO2A	FAM129B	KMO	MLPH	PPIB	SPDYE5	
ARHGEF37	COX11	FAM131A	KRCC1	MPRIP	PPP1R15B	SPG11	
ARHGEF40	CRACR2B	FAM186B	KRT18	MRPS34	PPP1R7	SPR	
ARHGEF5	CREB3L1	FAM221B	KRT19	MRPS5	PRDX2	SQOR	
ASPH	CROCCP3	FAM234A	KRT20	MSANTD2	PRDX5	ST6GALNAC1	
ASS1	CSKMT	FAM69A	LACTB2-AS1	MSC-AS1	PRKCSH	STEAP4	
ATG14	CSNK1D	FAM87B	LDHAL6B	MUC1	PROB1	STPG3	
ATP10B	CSNK2A1	FAT1	LGALS4	MUC13	PRRT2	TAGLN	
ATP1A1	CSTF2T	FBLIM1	LGALS9C	MUC17	PSMB8-AS1	TBC1D22A-AS1	
ATP2C2	CTAGE4	FCGBP	LIMA1	MUC3A	PSMC3IP	TDRD6	
ATP5F1B	CTAGE8	FDPSP2	LINC00537	MUC5B	PTPN2	TEX43	
ATP8B1	CTC1	FGL2	LINC00667	MUC5B-AS1	PTPRF	THAP3	
BAG1	CTCF	FLYWCH1	LINC00896	MXD3	PVR	THRIL	
BBIP1	CTSZ	FXR2	LINC01531	MYH15	RABL6	TIPARP	
BBS1	DAGLB	GALNT5	LINC02292	MYO6	RAD23A	TK1	
BDH1	DAZAP2	GBP1	LINC02352	NCAPH2	RALGPS1	TLCD2	
BFAR	DDT	GGA1	LOC100129083	NDUFA13	RAP1GAP	TMEM120B	
BNIP3	DDX21	GIT2	LOC100268168	NDUFA6	RASGRP1	TMEM183B	
BOK	DDX3X	GLYCTK-AS1	LOC100287808	NDUFAF6	RBCK1	TMEM30B	
BRI3	DDX51	GNRHR	LOC100506142	NFATC3	REG1A	TMEM8A	
BRPF3	DDX58	GOLGA3	LOC100507551	NFKBIA	RELL1	TMPO	
BTAF1	DDX6	GOLIM4	LOC100652768	NIPA1	RIF1	TMPRSS3	
BTF3	DEAF1	GPR82	LOC101927018	NOL4L	RNF126	TMSB4X	
C12orf73	DEFA5	GPRC5D	LOC101927377	NOXO1	RNF40	TNFSF10	
C1orf167	DEFA6	GRAMD2A	LOC101927770	NPHS2	ROGDI	TOP1	
C1orf174	DEK	GRIN1	LOC101927793	NSG1	RPL35	TRAF3	
C1QB	DLAT	GTF2A2	LOC101928371	NUTF2	RRBP1	TRAF3IP2-AS1	
C8orf59	DMBT1	GTF2E1	LOC101928844	NUTM1	S100A1	TRIP10	
CA1	DNAJB7	HDAC3	LOC102723313	OLFM4	SATB2	TSPOAP1-AS1	
CA12	DNAJC8	HEPHL1	LOC102724009	OR2A1	SELENOO	TTC28	
CALR	DNM1P41	HIF1A-AS2	LOC102724064	P2RY11	SFN	TUBB6	
