# Supplementary material for: High circulating elafin levels are associated with Crohn’s disease-associated intestinal strictures
Source: PLoS One. 2020 Apr 14;15(4):e0231796. doi: 10.1371/journal.pone.0231796 (PMC7156098; doi:10.1371/journal.pone.0231796)
Supplement: Attachment — Submitted filename: Reviewer comment Elafin study.docx [file pone.0231796.s009.docx]

02 February 2020

The Editor

PLoS One

Dear sir

Thank you for the privilege and opportunity to review the following study: **High circulating elafin levels are associated with Crohn’s disease-associated intestinal strictures.** This is indeed a timeous contribution in an otherwise difficult and complex medical problem.

My opinion is that the study should be published with minimal corrections to the manuscript.

Comments to the authors:

1. The title is appropriate and to the point and endeavours to prove the headline statement in the ensuing discussion.
2. In the Introduction section, I suggest a short succinct summary on the mechanisms involved in the fibrogenesis of Crohn’s disease and the link to elafin, clearly establishing the relationship.
3. Also in the Introduction on page 5 line 47 the following sentence should be amended to include the and…but the mechanism of this association is unknown AND has not yet been identified.
4. In the Materials and Methods section it must be made clear that there are 2 separate study groups (a) the serum sample group where only blood was taken and the (b) surgical group where colon and mesenteric fat is sampled. Also, that no blood was collected for the purposes of the study from (b). In the Inclusion and Exclusion criteria it should include whether active IBD at time of recruitment was a requirement for both the two groups mentioned above, especially for (a), or was the IBD quiescent at the time.
5. Further in the Materials and Methods section on page 6 line 47 the following sentence should be reviewed and amended: The healthy subjects did not have concurrent cancer, infection, obesity….. The control group in the surgical group (b) was patients with cancer requiring surgery! Maybe Inclusion and Exclusion criteria for the 2 study groups should be separated.
6. In the Results section the 2 study groups are discussed interchangeably creating the impression that all patients had all procedures. The importance here is that in the serum sample study according to supplementary Table 1 the Partial Mayo score for cohorts 1 and 2 were 3.9 and 4.2 while in the surgical group the colitis score was 6.8. Similarly, the HBI in CD was for the serum sample study was 3.6 and 4.2 for non-stricturing and stricturing disease respectively, while it was 7.4 and 5.0 in the surgical arm. This discrepancy should be explained and the extrapolation justified.
7. Can the authors please explain the discrepancy in the numbers ion Fig1D. The total number of CD patients in the serum study was 95. Where are the rest of the patients as results of only 65 are shown.
8. Finally, in the Results section, the mean age of the control group in surgical arm of the study was 20 years older than the IBD cohorts. Although the results suggest that this should not be of concern please corroborate this a reference as such.
9. The Discussion is generally well written and motivated, but I find some of the conclusions made are due to overinterpretation. From the data, serum elafin will not be a great discriminant for structuring CD as a stand-alone. Prospective testing will also increase the cost as the authors stated that elafin, at this point, is unable to predict future structuring. It should be stated that this may be an adjunct to currently available modes of investigation in CD in general.
10. Finally, the authors must give a schema of the clinical use of elafin in current clinical practice as a conclusion summary.

Thank you,

Sincerely

Erns Fredericks
